# Effects of the COVID-19 pandemic on early career neuroscientists

**DOI:** 10.1093/braincomms/fcac099

**Published:** 2022-05-02

**Authors:** Tara L. Spires-Jones

**Affiliations:** Centre for Discovery Brain Sciences, UK Dementia Research Institute, The University of Edinburgh, Edinburgh, UK

## Abstract

Our editor discusses the emerging effects of the COVID-19 pandemic on early career neuroscientists.

## Graphical Abstract

**Graphical Abstract:**
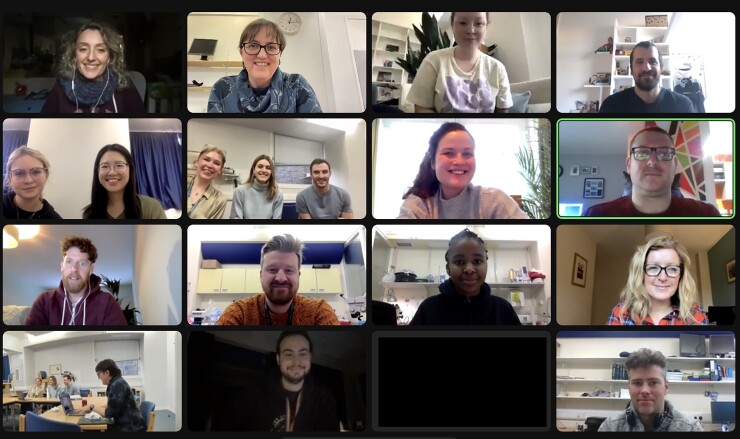

Welcome to Volume 4, issue 3 of *Brain Communications*. This week, I was very proud to watch my honours neuroscience undergraduate students present their dissertation research. They are doing cutting-edge experiments and contributing to papers and advancing our field. The presentations were top-notch, but this year and last year, I’ve noticed an uptick in the numbers of students struggling with anxiety, stress, and other mental health issues that are making it difficult for many to achieve their full potential. A few years ago, we would have a couple of students per cohort of around 60 who had difficulties, as many of us do at some point in our lives, and we do our best to help them with accommodations like extending deadlines. These numbers have steeply increased since the COVID-19 pandemic, which is not surprising. Due to lockdowns, these students have not been able to experience many typical experiences that increase resilience and build skills needed for a career in neuroscience. My observations of a few hundred students over the past 3 years are not an isolated occurrence. Chen and Lucock^1^ recently published a results of a survey of over 1000 students in England and found over 50% of respondents were experiencing anxiety and depression at levels above clinical cut offs. This is a worldwide phenomenon with studies in China, Canada and the US showing students were susceptible to the psychological effects of the pandemic including stress and anxiety.^2–4^

PhD students and postdocs also struggle with pandemic-related mental health issues and further were hit very hard by lab closures which prevented data collection during this critical phase of their careers.^5–8^ People have lost job opportunities and been unable to move to different countries for new positions. There is widespread concern that the long-term effects of the pandemic on researchers will fall disproportionately on women and widen the ‘gender gap’ in science.^9^ Our recent data looking at the gender of our authors in *Brain Communications* support this idea as we found decreasing proportions of women first authors from 2019 to 2021.^10^ This is also substantiated by many studies that find women report significantly more anxiety and depression during the pandemic than men.^1,3^

To address the negative effects of the pandemic, societies including the British Neuroscience Association,^11^ Federation of European Neurosciences and the Society for Neuroscience are advocating to governments to support early career neuroscientists. However, many agencies around the world have not been able or willing to do this, particularly in light of the financial crisis that was precipitated by the pandemic.

There is some help: charities that I work with, including Alzheimer’s research UK and the Scottish Dementia Research Consortium, have pivoted their funding models to support more early career researchers despite lower donations during the pandemic. And there are small silver-linings for early career researchers from behavioural changes during the pandemic. For example, giving talks online has become normalized and can be much easier for some with caring responsibilities. Similarly our ‘new normal’ in the lab is to have hybrid lab meetings which allow those of us who are in to see each other but those who need to work from home for any reason can still fully participate (see *Graphical Abstract* for a recent example). However, on balance, I worry about the negative effects of the pandemic on our field and particularly on students and early career researchers coming through the system now. If you have any thoughts about what we can and should do as a journal and as individual scientists, we’d love to hear from you either on Twitter @BrainComms or please get in touch about writing a field potential article on the topic.

The cover image comes from Sladky *et al*.^12^ and shows a pet dog with drug resistant epilepsy and MRI visualization of the brain for electrode implantation for treatment.
